# Crucial roles of intracellular cyclic di-GMP in impacting the genes important for extracellular electron transfer by *Geobacter metallireducens*

**DOI:** 10.1128/aem.00727-25

**Published:** 2025-06-04

**Authors:** Yongguang Jiang, Lin Sun, Lingyu Hou, Yidan Hu, Zhou Jiang, Yiran Dong, Hao Song, Liang Shi

**Affiliations:** 1 Department of Biological Sciences and Technology, School of Environmental Studies, China University of Geosciences 504988, Wuhan, China; 2 State Key Laboratory of Geomicrobiology and Environmental Changes, China University of Geosciences 12564, Wuhan, China; 3 State Environmental Protection Key Laboratory of Source Apportionment and Control of Aquatic Pollution, Ministry of Ecology and Environment, China University of Geosciences 12564, Wuhan, China; 4 Hubei Key Laboratory of Yangtze Catchment Environmental Aquatic Science, China University of Geosciences 12564, Wuhan, China; 5 College of Life and Health Sciences, Northeastern University 12434, Shenyang, China; University of Nebraska-Lincoln, Lincoln, Nebraska, USA

**Keywords:** cyclic di-GMP, *Geobacter metallireducens*, extracellular electron transfer, biofilms, Fe(III)-reduction, microbial fuel cells

## Abstract

To investigate the roles of intracellular c-di-GMP in bacterial extracellular electron transfer (EET), three *Geobacter metallireducens* strains with high (Gme-H), intermediate (Gme-C), and low (Gme-L) intracellular levels of c-di-GMP were constructed via the synthetic biology approach. Compared to Gme-C, Gme-H showed similar Fe(III) reduction rates, formed thicker biofilms on conductive and nonconductive surfaces, and produced more electricity, but showed delayed ability for electricity production. Gme-L formed thinner biofilms on nonconductive surfaces and reduced Fe(III)-citrate faster, but showed slower reduction of ferrihydrite in comparison to Gme-C. Although it produced electricity much faster, Gme-L produced less electricity and formed slightly less amounts of biofilms on anodes, as compared to Gme-C. The mRNA levels of multiple genes encoding *c*-type cytochromes (*c*-Cyts) and extracellular pilin protein PilA-N were differentially regulated in Gme-L or Gme-H in comparison to that in Gme-C. Expressions of the genes for PilA-N and extracellular *c*-Cyt Gmet2896 were increased by high c-di-GMP. Low c-di-GMP increased the gene expressions of the porin-cytochromes in the outer membrane. Further investigation also identified new c-di-GMP-regulated genes directly involved in the EET of *G. metallireducens*, such as those for the *c*-Cyts of extracellular Gmet0601, the periplasmic Gmet1703 and Gmet1809 on the cytoplasmic membrane, as deletions of these genes impaired bacterial reductions of extracellular ferrihydrite and anode. Thus, intracellular c-di-GMP impacted multiple genes of *G. metallireducens* whose protein products might transfer electrons from the cytoplasmic membrane, through the periplasm, across the outer membrane to and in the extracellular environment.

**IMPORTANCE:**

Bis-(3′−5′)-cyclic dimeric guanosine monophosphate (c-di-GMP) is ubiquitous in bacterial cells where it regulates a variety of bacterial processes, which range from biofilm formation, bacterial virulence to cell cycle progression. However, its role in regulating bacterial extracellular electron transfer is much less characterized. This investigation shows the crucial roles of intracellular c-di-GMP in impacting the extracellular electron transfer of the Gram-negative bacterium *Geobacter metallireducens*. The gene expressions of the multiheme *c*-type cytochromes in the bacterial cytoplasmic membrane, periplasm, outer membrane, and extracellular environment, as well as the gene expression of extracellular pilin protein PilA-N, are all impacted by c-di-GMP. Although how it impacts the expression of these genes is currently unclear, c-di-GMP affects the entire extracellular electron transfer process of *G. metallireducens* from the cytoplasmic membrane, through the periplasm and across the outer membrane to and in the extracellular environment.

## INTRODUCTION

As a second messenger, bis-(3′−5′)-cyclic dimeric guanosine monophosphate (c-di-GMP) is ubiquitous in bacterial cells where it regulates a variety of bacterial processes, which range from biofilm formation, bacterial virulence, to cell cycle progression (1–3). The intracellular level of c-di-GMP is controlled by the c-di-GMP-synthesizing enzymes such as diguanylate cyclases (DGCs) with GGDEF domains, and c-di-GMP-degrading enzymes, such as phosphodiesterases (PDEs) with either EAL or HD-GYP domains (1). The roles of c-di-GMP in intracellular signaling can be local or global (4). Specific DGCs, PDEs, and c-di-GMP effector components may form larger complexes in the local signaling system, where c-di-GMP is generated right next to its effector (4). For global signaling, cellular c-di-GMP level is controlled by DGCs antagonized by PDEs responding to external or cellular conditions, and c-di-GMP reaches its effector by diffusion (4).

Bacterial extracellular electron transfer (EET) occurs under anoxic conditions. For example, the dissimilatory Fe(III)-reducing bacterium *Geobacter sulfurreducens* respires on solid-phase Fe(III) oxides and anodes for anaerobic growth via EET (5, 6). Electron transfer to the anode generates electricity in microbial fuel cells. In addition, *G. sulfurreducens* exchanges electrons with the cells of other bacterial species via direct interspecies electron transfer (DIET) (7, 8). Previous results revealed that *G. sulfurreducens* contained 29 putative diguanylate cyclases with GGDEF domains (9). Our previous results discovered that c-di-GMP differentially regulated the EET genes of *G. sulfurreducens* (10). High levels of c-di-GMP increased the electricity production of *G. sulfurreducens* via increased thickness of the bacterial biofilms on anodes and increased expression of the genes encoding periplasmic cytochromes involved in bacterial EET, such as multiheme *c*-cytochrome (*c*-Cyt) PpcA (10). Although a low level of c-di-GMP decreased the thickness of bacterial biofilms on anodes, it also increased the electricity production of *G. sulfurreducens* via increased expression of some genes crucial for bacterial EET, such as *omcE*, *omcS*, *omcZ,* and *pilA-N* (10). OmcE, OmcS, and OmcZ are multiheme *c*-Cyts, and PilA-N is the N-terminal portion of pilin protein PilA. They can form or be part of extracellular nanofilaments critical for EET (11–20).

*G. metallireducens* is an environmentally and biotechnologically important bacterium (21). Similar to *G. sulfurreducens*, EET is also key to the physiology of *G. metallireducens* (22). The PilA-N and multiheme *c*-Cyts are also directly involved in the EET of *G. metallireducens* (22–26). Although some of these *c*-Cyts have homologs in other *Geobacter* species, recent investigations suggested that these homologs might function differently (27–31). Twenty putative DGCs containing GGDEF domains were predicted in the genome of *G. metallireducens*, indicating that c-di-GMP may play an important role in the signaling system of this bacterium (31). In addition to c-di-GMP, cyclic AMP-GMP (also referenced as cGAMP) is also a cyclic dinucleotide that is synthesized by hybrid promiscuous GGDEF enzymes (32). Similar to c-di-GMP, cGAMP is also a second messenger in intracellular signaling (33). Previous investigations found that cGAMP was involved in controlling exoelectrogenesis of *G. metallireducens* by binding to GEMM-I riboswitches (9, 34). However, little is known about how the EET genes in *G. metallireducens* are regulated by c-di-GMP.

In this investigation, three *G. metallireducens* (Gme) strains with different intracellular levels of c-di-GMP were constructed: Gme-L with a low level of intracellular c-di-GMP, Gme-C with an intermediate level of intracellular c-di-GMP, and Gme-H with a high level of intracellular c-di-GMP. These strains were compared in biofilm formation on a nonconductive surface, Fe(III), and anode reductions and transcriptomes during anode reduction. Further investigation of the c-di-GMP-impacted genes identified new genes involved in EET of *G. metallireducens*. The results from this investigation revealed crucial roles of c-di-GMP in EET of *G. metallireducens*.

## RESULTS

### Bacterial strains of different levels of intracellular c-di-GMP

**Fig 1:**
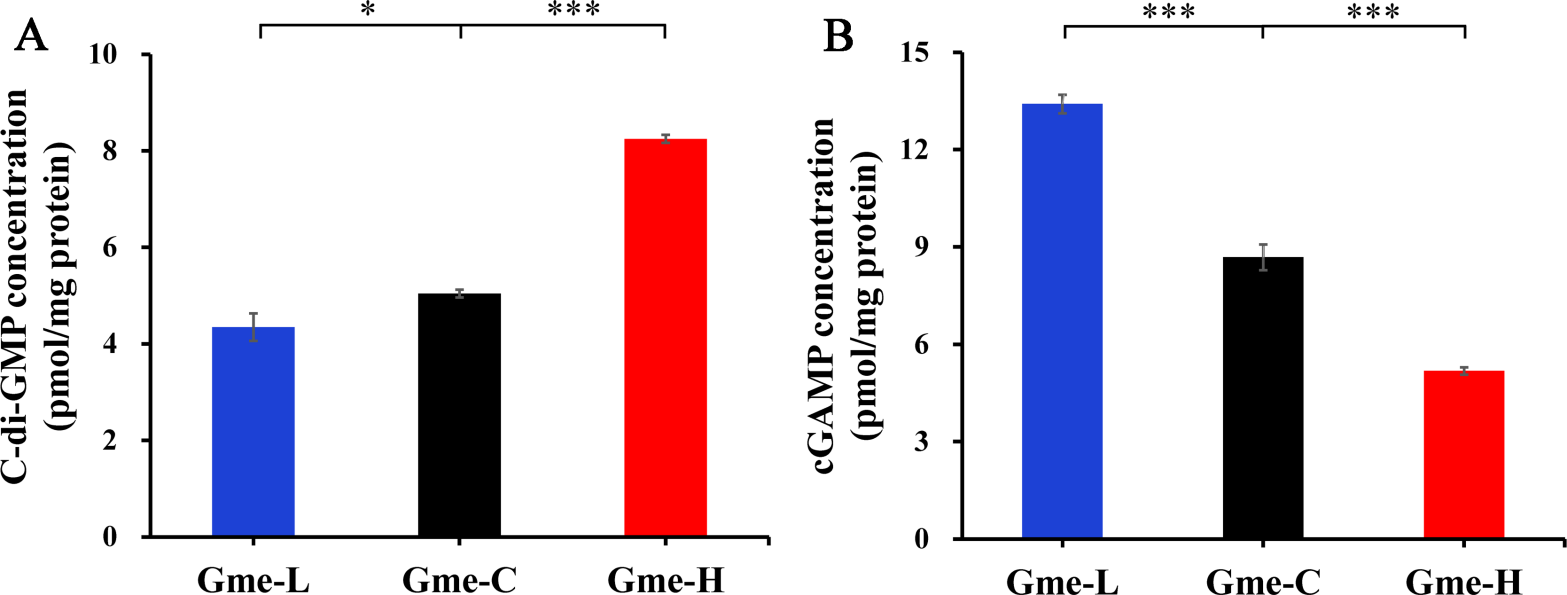
Intracellular levels of c-di-GMP (**A**) and cGAMP (**B**) in *G. metallireducens* (Gme) strains grown under Fe(III)-citrate respiring conditions. All results are reported as mean and standard deviation (*n* = 3). *, 0.01 < *P* < 0.05; **, 0.001 < *P* < 0.01, ***P < 0.001. Gme-L, strain with the plasmid containing a c-di-GMP-degrading gene; Gme-C, strain with the empty vector; Gme-H, strain with the plasmid containing a c-di-GMP-synthesizing gene.

To evaluate the impacts of c-di-GMP on the EET of *G. metallireducens*, the previously made pYhjH with a c-di-GMP-degrading gene, pYedQ with a c-di-GMP-synthesizing gene, and the empty vector pYYDT were introduced into *G. metallireducens* individually (10). The resulting strains Gme-L with pYhjH, Gme-C with pYYDT, and Gme-H with pYedQ were first compared for their intracellular levels of c-di-GMP under Fe(III)-citrate respiring conditions. The amount of c-di-GMP detected in Gme-H was 8.25 ± 0.08 pmoL/mg protein (*n* = 3) that was significantly (*P* < 0.001) higher than that of the control strain Gme-C (5.04 ± 0.08 pmoL/mg protein, *n* = 3) (Fig. 1A). The amount of c-di-GMP detected in Gme-L was 4.35 ± 0.29 pmoL/mg protein (*n* = 3) that was significantly (*P* < 0.05) lower than that of Gme-C (Fig. 1A). The intracellular levels of cGAMP, however, decreased significantly in the order of Gme-L (13.41 ± 0.28 pmoL/mg protein, *n* = 3) >Gme C (8.68 ± 0.40 pmoL/mg protein, *n* = 3) >Gme H (5.18 ± 0.12 pmoL/mg protein, *n* = 3) (Fig. 1B).

### Biofilm formation on a nonconductive surface

**Fig 2:**
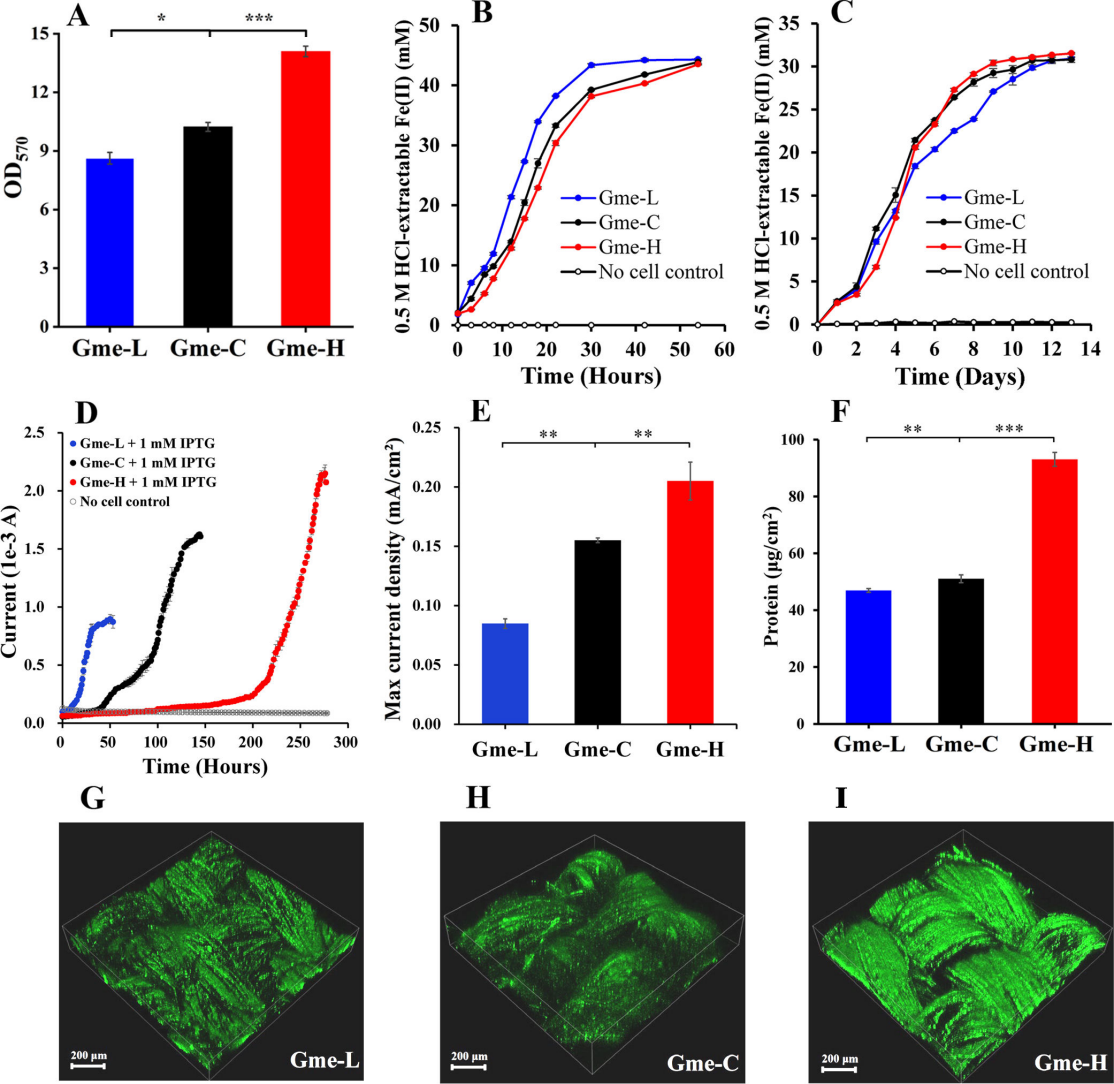
Characterization of the *G. metallireducens* strains with different intracellular levels of c-di-GMP. (**A**) Biofilm formation. Shown are the absorbance of crystal violet (OD_570_) extracted from stained biofilms on non-conductive surfaces after 60 hours of growth. All results are reported as mean and standard deviation (*n* = 4). (**B**) Fe(III)-citrate reduction. (**C**) Ferrihydrite reduction. (**D**) Electricity production. (**E**) Maximum current density. (**F**) Amounts of proteins detected in the biofilms on anodes during maximum electricity production. All results are reported as mean and standard deviation (*n* = 3). For points with no error bar, the error was smaller than the size of the symbol. *0.01 < *P* < .05; **0.001 < *P* < .01, ***P < 0.001. (G–I) CLSM images of biofilms on anodes for the bacterial strains Gme-L (**G**), Gme-C (**H**), and Gme-H (**I**).

All tested strains formed biofilms on the nonconductive surface of 24-well cell culture plates. For all strains, the amounts of biofilms increased during 60 hours and decreased afterward (Fig. S1). The absorbance (OD_570_) maximums of extracts from crystal violet-stained biofilms decreased in the order of Gme-H >Gme C >Gme L (Fig. 2A).

### Fe(III) reduction and electricity production

Compared to that of Gme-C and Gme-H, Fe(III)-citrate reduction by Gme-L was faster during the initial 12 hours and plateaued earlier at the 30th hour after reduction (Fig. 2B). The Fe(III)-citrate reduction traces of Gme-H and Gme-C are close, and Gme-H was only slightly slower than Gme-C at an early stage of reduction. All strains reduced similar amounts of Fe(III)-citrate at the end of reduction (Fig. 2B). Gme-L reduced ferrihydrite more slowly than Gme-C, although they reduced similar amounts of ferrihydrite during the first 12 days (Fig. 2C). Compared to that of Gme-C, ferrihydrite reduction by Gme-H was slower during the initial 3 days, but it plateaued earlier at the 10th day after reduction (Fig. 2C).

The electricity production by Gme-L was detectable after 10 hours of growth and plateaued after 48 hours of growth (Fig. 2D). The electricity production by Gme-C was detectable after 45 hours of growth and plateaued after 108 hours of growth (Fig. 2D). The electricity production by Gme-H was detectable after 192 hours of growth and plateaued after 256 hours of growth (Fig. 2D). The maximum current density of Gme-H was 0.189 ± 0.002 mA/cm^2^ (*n* = 3) that was significantly (*P* < 0.01) higher than that of Gme-C (0.159 ± 0.005 mA/cm^2^) (Fig. 2E). The maximum current density of Gme-L was 0.118 ± 0.008 mA/cm^2^ (*n* = 3) that was significantly (*P <* 0.01) lower than that of Gme-C (Fig. 2E). Biofilm biomass on the anode surface was detected as protein amounts after the electricity production reached maximum. The amounts of proteins decreased in the order of Gme-H >Gme C >Gme L (Fig. 2F). Examinations with a confocal laser scanning microscope (CLSM) also showed more biofilms formed on the anode surface by Gme-H than by Gme-C or Gme-L (Fig. 2G through I). The concentrations of c-di-GMP and cGAMP in the planktonic cells and biofilms of Gme-L, Gme-C, and Gme-H that were grown in anode chambers increased and decreased in the same order as those in the cells grown under Fe(III)-citrate respiring conditions, respectively (Fig. S2).

### Transcriptomic and immunoblot analyses

**Fig 3:**
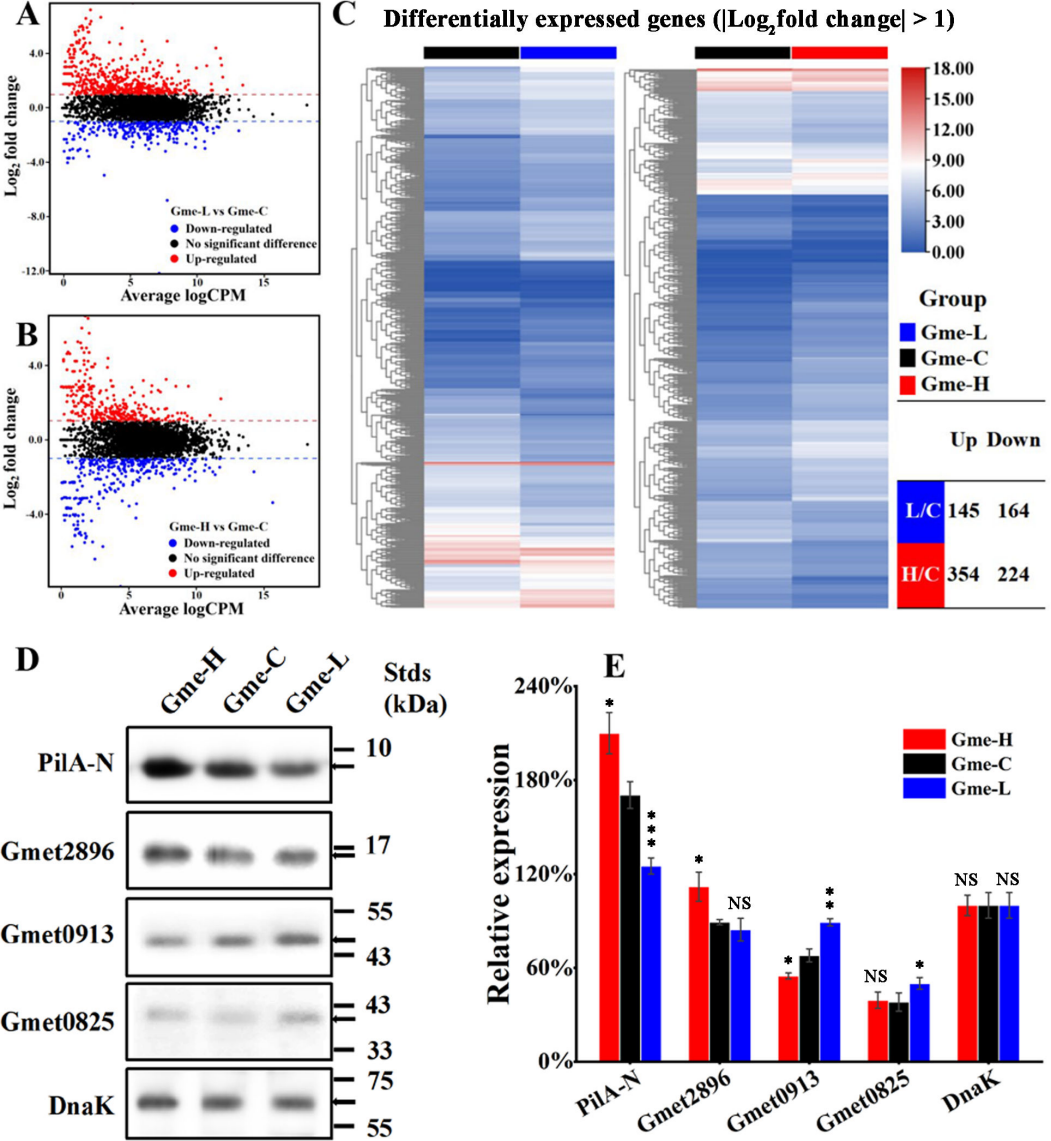
Transcriptomic and immunoblot analyses of the biofilms of *G. metallireducens* strains with different intracellular levels of c-di-GMP on anodes during maximum electricity production. (**A**) Mean-difference (MD) plots for the comparison of differentially expressed genes between Gme-L and Gme-C. (**B**) MD plots for the comparison of differentially expressed genes between Gme-H and Gme-C. (**C**) The heatmaps of differentially expressed genes in Gme-L and Gme-H. (**D**) PilA-N, Gmet2896, Gmet0825, Gmet0913, and DnaK proteins were detected in the biofilms on anodes with their respective antibodies. The migration positions of standard proteins (Stds) in kilodaltons (kDa) are shown on the right. (**E**) The relative abundance of PilA-N, Gmet2896, Gmet0825, Gmet0913, and DnaK proteins in the biofilms on anodes. NS, *P* > 0.05; *0.01 < *P* < 0.05; **0.001 < *P* < 0.01, ***P < 0.001.

To investigate c-di-GMP-regulated genes, the transcriptomes of the Gme-L, Gme-C, and Gme-H biofilms formed on the anode surfaces were compared with RNAseq after electricity production reached maximum. A total of 3,679 genes were identified (Fig. S3A). As shown in principal component analysis, the transcriptomic profiles of the Gme-L, Gme-C, and Gme-H biofilms were distinct (Fig. S3B). Compared to that in Gme-C, the mRNA levels of 145 genes increased and those of 164 genes decreased in Gme-L (Fig. 3A and C left panel). Similarly, the mRNA levels of 354 genes increased and those of 224 genes decreased in Gme-H, as compared to Gme-C (Fig. 3B and C right panel).

Notably, distinct transcription changes were shown for the *c*-Cyts genes in Gme-L or Gme-H as compared to Gme-C, including those involved in EET (Table S1). The mRNA level of the *Gmet1399* gene encoding pilin protein PilA-N increased in Gme-H, as compared to that in Gme-C (Table S1). Immunoblot analyses also showed that PilA-N and Gmet2896 levels in Gme-H were higher than those in Gme-C (Fig. 3D and E). Compared to that in Gme-C, the Gmet0825 and Gmet0913 levels in Gme-L increased, but the PilA-N level was lower in Gme-L than in Gme-C. The DnaK levels in these strains were similar (Fig. 3D and E).

Transcription changes were also observed for several genes predicted to be controlled by c-di-GMP and cGAMP riboswitches in Gme-L and Gme-H (Table S2). *Gmet1191* and *Gmet1703* were predicted to be activated by cGAMP (9, 34) and were significantly upregulated (Log_2_FC > 1, *P* < 0.05) in Gme-L with a higher level of cGAMP than Gme-C (Table S2). However, although some genes (e.g., *Gmet0972*, *Gmet1087*, *Gmet1703*, *Gmet3485*, *Gmet3486,* and *Gmet3487*) were predicted to be activated by cGAMP, they were significantly upregulated (Log_2_FC > 1, *P* < 0.05) in Gme-H with lower levels of cGAMP than Gme-C (Table S2).

### Characterization of EET genes regulated by c-di-GMP

**Fig 4:**
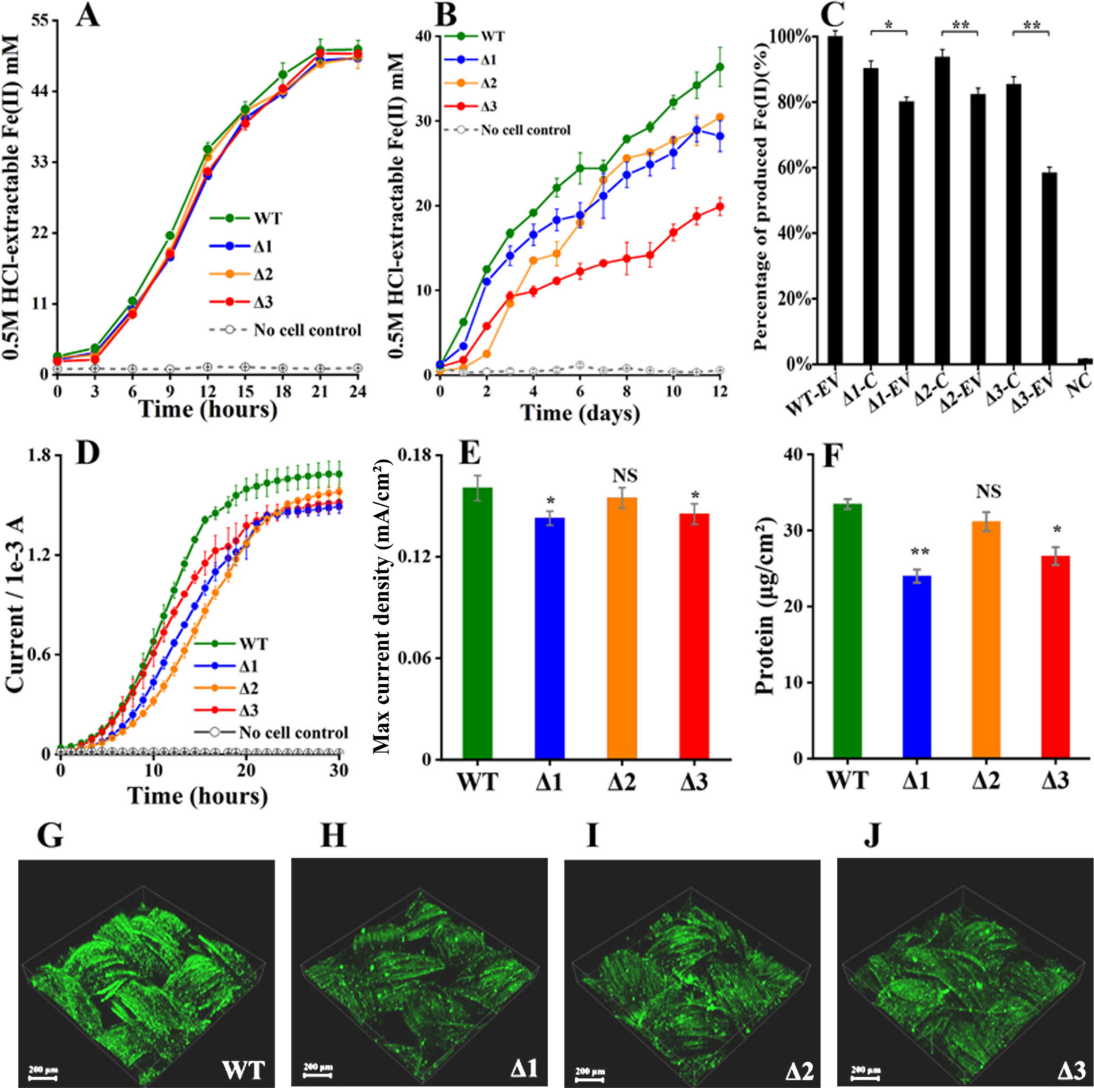
Characterizations of the newly identified genes regulated by intracellular c-di-GMP in *G. metallireducens*. (**A**) Fe(III)-citrate reduction. (**B**) Ferrihydrite reduction. (**C**) Complementation. Shown are the results on the 12th day after the reduction of ferrihydrite. EV, empty vector; C, complement; NC, no cell control. The amount of 100% Fe(II) produced was 35.48 ± 0.45 mM (*n* = 3). (**D**) Electricity production. (**E**) Maximum current density. (**F**) Amounts of proteins detected in the biofilms on anodes during maximum electricity production. All results are reported as mean and standard deviation (*n* = 3). For points with no error bar, the error was smaller than the size of the symbol. Asterisks indicate significance levels of difference between mutant and wild-type strains. NS, *P* > 0.05; *0.01 < *P* < .05; **0.001 < *P* < .01, ***P < 0.001. (G–J) Biofilms on anodes of wild type (WT), Δ*Gmet0601* (Δ1), Δ*Gmet1703* (Δ2), and Δ*Gmet1809* (Δ3) after 30 hours of growth.

Transcriptomic analyses revealed that the genes for *c*-Cyts Gmet0601, Gmet1703, and Gmet1809 were impacted by c-di-GMP (Table S1). To evaluate their roles in the EET of *G. metallireducens*, *Gmet0601*, *Gmet1703,* and *Gmet1809* were individually deleted. Deletions of these genes had no impact on bacterial reduction of Fe(III)-citrate (Fig. 4A), but diminished the rates and amounts of ferrihydrite reduction within 12 days (Fig. 4B; Fig. S4). Compared to that with an empty vector, the mutants complemented with their respective genes all showed increased ability to reduce ferrihydrite (Fig. 4C; Fig. S4). Compared to the wild type (WT) of *G. metallireducens*, the gene-deletion mutants all showed the delayed and/or reduced ability to produce electricity on anodes (Fig. 4D). The maximum current density of WT was higher than that of Δ*Gmet0601* and Δ*Gmet1809*, but was similar to that of Δ*Gmet1703* (Fig. 4E). Similarly, the amounts of proteins in the WT biofilms on the anodes were higher than those of Δ*Gmet0601* and Δ*Gmet1809* after electricity production plateaued, but were similar to those of Δ*Gmet1703* (Fig. 4F). CLSM examinations of the biofilms on the anodes showed similar results (Fig. 4G through J). The predicted subcellular localizations were extracellular for Gmet0601, periplasmic for Gmet1703, and on the cytoplasmic membrane for Gmet1809.

## DISCUSSION

Introduction of a bacterial c-di-GMP-synthesizing gene into *G. metallireducens* increased the intracellular c-di-GMP level and thickness of the biofilms on a nonconductive surface. On the contrary, the introduction of a bacterial c-di-GMP-degrading gene into *G. metallireducens* decreased the intracellular c-di-GMP level and thickness of the biofilms on a nonconductive surface. These results are consistent with previous findings in *G. sulfurreducens* (10).

This study showed for the first time that decreased levels of intracellular c-di-GMP promoted extracellular reduction of Fe(III)-citrate but delayed that of ferrihydrite by *G. metallireducens*, while increased levels of intracellular c-di-GMP had minimal impact on the reductions of Fe(III)-citrate and ferrihydrite. Most importantly, the impacts of low intracellular levels of c-di-GMP on electricity production by *G. metallireducens* also differed substantially from those of high intracellular levels of c-di-GMP. Although it shortened the time for initiating electricity production, a low intracellular level of c-di-GMP lowered the maximum electricity production. The impact of a low intracellular level of c-di-GMP on biofilm formation on the anode was minimal. High intracellular levels of c-di-GMP, however, delayed electricity production but improved biofilm formation on anodes and increased the maximum electricity production. Thus, intracellular c-di-GMP differentially regulates the EET of *G. metallireducens*.

It should be noted that the regulation of the EET in *G. metallireducens* by intracellular c-di-GMP differed from that in *G. sulfurreducens* (10). In *G. sulfurreducens*, a low intracellular level of c-di-GMP significantly decreased the biofilm formation on the anodes, but substantially increased the maximum electricity production (10). Furthermore, the impact of low intracellular levels of c-di-GMP on the time for initiating electricity production in *G. metallireducens* was more pronounced than that in *G. sulfurreducens* (10). Similarly, high intracellular levels of c-di-GMP in *G. metallireducens* delayed electricity production longer than those in *G. sulfurreducens* (10). These results suggest that the molecular mechanisms used by c-di-GMP for regulating the EET of *G. metallireducens* may be different from that in *G. sulfurreducens*. Consistent with this suggestion, the results from this investigation showed that high intracellular c-di-GMP increased the expression of the *pilA-N* gene in *G. metallireducens,* and low intracellular c-di-GMP decreased the expression of the *pilA-N*. This is in sharp contrast to that in *G. sulfurreducens*, in which low intracellular c-di-GMP increased *pilA-N* expression and high intracellular c-di-GMP decreased *pilA-N* expression (10).

Notably, the expression of the gene for extracellular *c*-Cyt Gmet2896 was also increased by high intracellular c-di-GMP. Similar to PilA-N, Gmet2896 was also directly involved in the EET of *G. metallireducens* (23–25, 35). Gmet2896 shared sequence similarity with OmcE of *G. sulfurreducens* (24). OmcE formed conductive filaments and was involved in the EET of *G. sulfurreducens* (17, 18, 36). In *G. sulfurreducens*, *omcE* expression was also co-regulated with *pilA-N* expression by low intracellular c-di-GMP (10). Thus, similar to OmcE in *G. sulfurreducens*, Gmet2896 in *G. metallireducens* may also form the conductive filaments.

This investigation also identified other c-di-GMP-regulated genes for the EET of *G. metallireducens. G. metallireducens* possessed three *pcc* gene clusters *Gmet0825-0828*, *Gmet0908-0910,* and *Gmet0911-0913* for EET across the bacterial outer membrane (29). Deletions of *Gmet0910*, *Gmet0912,* or *Gmet0913* all negatively impacted the DIET from *G. metallireducens* to other microorganisms (22). This investigation revealed that expressions of *Gmet0825* and *Gmet0913* were increased by low intracellular c-di-GMP. Thus, the Pcc complexes on the outer membrane of *G. metallireducens* are regulated by low intracellular c-di-GMP. It should also be noted that how intracellular c-di-GMP regulates the expression of these genes is currently unknown.

Previous investigations showed that expressions of *Gmet0601*, *Gmet1703,* and *Gmet1809* were increased during coculture of *G. metallireducens* with other microorganisms (22, 37). This investigation, for the first time, provided the direct evidence for the involvement of these *c*-Cyts in the EET of *G. metallireducens*. The predicted subcellular locations were extracellular for Gmet0601, periplasmic for Gmet1703, and on the cytoplasmic membrane for Gmet1809. Gmet1809 was also predicted to be the subunit of alternative complex III (ACIII) that is involved in quinol oxidation on the cytoplasmic membrane (38). Thus, multiheme *c*-Cyts Gmet0601, Gmet1703, and Gmet1809 are probably involved in electron transfer in the extracellular environment, the periplasm, and the cytoplasmic membrane, respectively.

The cGAMP level was upregulated and downregulated in Gme-L and Gme-H, respectively, which shows an inverse relationship between the concentrations of c-di-GMP and cGAMP. In *G. metallireducens* GS-15, the diguanylate cyclase Gmet1914 was found to be a hybrid promiscuous (Hypr) GGDEF enzyme that might synthesize cGAMP (32). The mRNA level of Gmet1914 is not significantly changed in Gme-L and Gme-H, as compared to that in Gme-C. It was reported that c-di-GMP inhibited the activity of cGAMP synthase GacB of *Myxococcus xanthus*, which is also a Hypr GGDEF enzyme (39). Therefore, the observed inverse relationship between the concentrations of c-di-GMP and cGAMP may be attributed to inhibition of Gmet1914 or other cGAMP synthases by c-di-GMP.

It is interesting that several genes predicted to be activated by cGAMP were upregulated in Gme-H with a lower cGAMP level. Although the exact reason for this observed discrepancy is currently unknown, these genes may be upregulated by c-di-GMP that also binds to GEMM-I riboswitches (9, 34) and is abundant in Gme-H. In addition, cautions must be excised in analyses of transcriptomic data of the biofilms on electrodes, as other factors, such as biofilm thickness and diffusion in biofilm, may also affect biofilm transcriptomes.

In conclusion, this investigation identifies a group of EET genes of *G. metallireducens* whose expressions are regulated by the intracellular c-di-GMP level. Proteins encoded by these EET genes are strategically positioned in the cytoplasmic membrane, periplasm, and outer membrane, which may form the pathways for EET. Thus, intracellular c-di-GMP plays crucial roles in regulating the EET of *G. metallireducens* from the cytoplasmic membrane, through the periplasm, and across the outer membrane to and in the extracellular environment. Alteration of intracellular c-di-GMP level via the synthetic biology approach is a unique way to identify the genes directly involved in bacterial EET. The results of this investigation also reveal the new molecular components directly involved in bacterial EET.

## MATERIALS AND METHODS

### Bacterial strains and cultural condition

Originally purchased from American Type Culture Collection (Manassas, VA, USA) (Table S3), *Geobacter metallireducens* GS-15 (ATCC 53774) was routinely cultured at 30 ℃ in the bicarbonate-buffered medium in which 20 mM acetate served as an electron donor and 50 mM Fe(III)-citrate served as an electron acceptor (25). Gme-L, Gme-C, and Gme-H were made by introducing previously constructed plasmids pYhjH, pYYDT, and pYedQ separately into *G. metallireducens* (Table S3) (10). YhjH and YedQ were recently renamed to PdeH and DgcQ, respectively (40). The constructed Gme-L, Gme-C, and Gme-H were cultured at 30°C in the bicarbonate-buffered medium supplemented with 100 µg/mL kanamycin and 1 mM IPTG. Five milliliter of cell culture was collected when half the amounts of Fe(III)-citrate were reduced. Cells were harvested by centrifugation for biochemical analysis. Bacterial inoculation and sampling were conducted in an anaerobic chamber (N_2_/CO_2_, 80%/20%, vol/vol, Coy Laboratory Products, Inc, Grass Lake, MI, USA). Acetate, Fe(III)-citrate, and other chemicals were purchased from Sinopharm Chemical Reagent Co., Ltd (Shanghai, China). Kanamycin was purchased from Guangzhou Saiguo Biotech Co., Ltd (Guangzhou, China), and IPTG was purchased from Biosharp Life Sciences (Hefei, China).

### Gene deletion

The gene deletion mutants were constructed in accordance with previously published procedures (25). After verification with PCR and DNA sequencing of deletion regions, the mutants were complemented with their respective genes. An empty vector was introduced into the mutants as controls. Strains containing vectors were cultured in the bicarbonate-buffered medium supplemented with 100 µg/mL kanamycin. Tables S3 and S4 list the mutants constructed and plasmids and oligo-primers used in this investigation, respectively.

### Protein, c-di-GMP, and cGAMP measurements

Bacterial cells were lysed using the EX2521 Bacterial Protein Extraction Kit (SolarBio, China) according to the manufacturer’s protocol. The resulting solution was used for measurement of proteins, c-di-GMP, and cGAMP. The amounts of proteins were measured using the Pierce BCA Protein Assay Kit (Thermo Fisher Scientific, USA). Cyclic di-GMP and cGAMP ELISA Kits (Meimian Bio, China) were used to detect c-di-GMP and cGAMP.

### Biofilm assay on a nonconductive surface

*G. metallireducens* strains were grown in 24-well cell culture plates with an initial optical density of 0.1 at 600 nm under Fe(III)-respiring conditions. Each strain was inoculated into 24 wells. At predetermined time points, cultures from four wells were collected and used for biofilm measurement. The amounts of biofilms formed on the well surface were determined with the crystal violet staining method (10, 18). Crystal violet was purchased from Shanghai Maikelin Biochemical Technology Co., Ltd (Shanghai, China).

### Fe(III)-citrate and Ferrihydrite reduction

Fe(III)-citrate reduction was monitored during the growth of *G. metallireducens* as described in a previous study (41). Two-line ferrihydrite was prepared by following a previously described method (18, 42). Ferrihydrite reduction was performed at 30°C in the bicarbonate-buffered medium in which Fe(III)-citrate was replaced with ferrihydrite. For strains with vectors, 100 µg/mL kanamycin and 1 mM IPTG were supplemented in the medium. At predetermined time points, 1 mL of culture was collected and centrifuged at 5,000 rpm for 2 min. The Fe(II) in the supernatants was determined with the ferrozine assay (43) and a UV–Vis spectrometer (Thermo Fisher Scientific, USA).

### Electricity production

Electricity production was measured in microbial fuel cells (MFC) with two chambers and three electrodes (10). The anode chamber was filled with 140 mL of anoxic medium. For strains with vectors, 100 µg/mL kanamycin and 1 mM IPTG were supplemented in the anolyte. Acetate (20 mM) was supplemented as an electron donor. Carbon cloth (3 cm × 3.5 cm) served as the anode and the sole electron acceptor. Anode voltage was set as +100 mV versus Ag/AgCl. A piece of platinum mesh (1.5 cm × 1.5 cm) served as the cathode. The catholyte contained 50 mM sodium chloride without removal of oxygen. A CHI 1000C electrochemical workstation (CHI, Shanghai, China) with an Ag/AgCl reference electrode was used for current output measurement. For each strain, six replicates were performed. After electricity production plateaued, the biofilms formed on the anode surface were examined with a confocal laser scanning microscope (Leica Microsystems CMS GmbH, Germany). To measure the amounts of proteins, c-di-GMP and cGAMP in the anode chamber, planktonic cells in the anolyte were harvested by centrifugation. Biofilm on the anodes was collected by cutting the carbon cloth into pieces and suspending them in the lysis buffer of the SolarBio EX2521 Kit directly.

### Transcriptomic analyses

After electricity production plateaued, the biofilms formed on the anode surface were also collected for RNA isolation with a HiPure Universal RNA Mini Kit (Magen, Guangzhou, China) (10). Biofilms from triplicate MFCs were mixed as one sample. Two samples were used for RNA extraction and sequencing for each strain. The Epicentre Ribo-Zero rRNA Removal Kit (Illumina, San Diego, CA, USA) was used to remove rRNA, and the NEBNext Ultra II Directional RNA Library Prep Kit (Illumina) was used to construct cDNA libraries that were sequenced at the Guangdong Magigene Biotechnology Co. Ltd. China (Guangdong, China). The sequencing data were trimmed with Trimmomatic (44). rRNA reads were removed with Bowtie2 combined with RefSeq and Rfam 14 (45–47). Bowtie2 was also used to compare the non-rRNA reads with the genome of *G. metallireducens* (NC_007517.1). The gene abundances were normalized with the TPM method (48). The results of principal component analysis (PCA) of Bray-Curtis distance and MD plots methods were visualized with RStudio. The differentially expressed genes were analyzed with edgeR (49). Transcriptomic data were deposited in the NCBI database with BioProject ID PRJNA1132199. An additional file showing the transcriptional changes of genes in Gme-L and Gme-H as compared to Gme-C was provided.

### Immunoblot analyses

Table S5 lists the polypeptides used for antibody production in this study. The prepared antibodies were characterized by following the procedures described before (10, 50). Antibodies specific for PilA-N, Gmet2896, and Gmet0825 were successfully prepared (Fig. S5). Antibodies specific for Gmet0913 and DnaK were prepared in a previous study (51). The proteins isolated from the biofilms on the anodes were analyzed with the produced antibodies (10, 50). Goat anti-rabbit IgG-HRP was purchased from TransGen Biotech (Beijing, China). Pageruler prestained protein ladder was purchased from Thermo Fisher Scientific. The super-sensitive ECL luminescence reagent was purchased from Meilunbio (Dalian, China). Azure C300 (Azure Biosystems, Inc., Dublin, CA, USA) was used to detect the antibody-protein interactions.

### Prediction of subcellular localization of proteins

Subcellular localizations of proteins were predicted with Cell-Ploc 2.0 and PSORTb 3.0 (52, 53).

### Statistical analyses

All the data were obtained from at least three biological replicates. The values are expressed as means ± standard deviations. Student’s *t* test was used for comparing groups.

## Data Availability

The transcriptomic data from this investigation were submitted to the NCBI BioProject Database with BioProject ID PRJNA1132199.

## SUPPLEMENTAL MATERIAL

Supplemental material aem.00727-25-s0001.docx Tables S1 to S5 and Fig. S1 to S5.

Additional supplemental tables aem.00727-25-s0002.xlsx Tables S6 and S7.

ASM does not own the copyrights to Supplemental Material that may be linked to, or accessed through, an article. The authors have granted ASM a non-exclusive, world-wide license to publish the Supplemental Material files. Please contact the corresponding author directly for reuse.
