# Supplementary material for: Crucial roles of intracellular cyclic di-GMP in impacting the genes important for extracellular electron transfer by Geobacter metallireducens
Source: Appl Environ Microbiol. 2025 Jun 4;91(7):e00727-25. doi: 10.1128/aem.00727-25 (PMC12285248; doi:10.1128/aem.00727-25)
Supplement: Supplemental material — Tables S1 to S5 and Fig. S1 to S5. [file aem.00727-25-s0001.docx]

**The crucial roles of intracellular cyclic di-GMP in impacting the genes important for extracellular electron transfer by *Geobacter metallireducens***

Yongguang Jiang^1*^, Lin Sun^1#^, Lingyu Hou^1#^, Yidan Hu^1^, Zhou Jiang^1^, Yiran Dong^1,2,3,4^, Hao Song^5^, Liang Shi^1,2, 3,4*^

^1^ Department of Biological Sciences and Technology, School of Environmental Studies, China University of Geosciences, Wuhan, China

^2^ State Key Laboratory of Geomicrobiology and Environmental Changes, China University of Geosciences, Wuhan, China

^3^ State Environmental Protection Key Laboratory of Source Apportionment and Control of Aquatic Pollution, Ministry of Ecology and Environment, China University of Geosciences, Wuhan, China

^4^ Hubei Key Laboratory of Yangtze Catchment Environmental Aquatic Science, China University of Geosciences, Wuhan, China

^5^ College of Life and Health Sciences, Northeastern University, Shenyang, China

5 tables

5 figures

14 pages

Table S1 Transcriptional changes of *c*-type cytochrome genes and *pilA-N* in Gme-L and Gme-H as compared to Gme-C. FC, fold change. *, 0.01 < *P* < 0.05; **, 0.001 < *P* <0.01; ***, *P* < 0.001.

| **Locus ID** | **Annotation** | **Location** | **Log_2_FC**  **(Gme-L vs Gme-C)** | **Log_2_FC**  **(Gme-H vs Gme-C)** |
| --- | --- | --- | --- | --- |
| Gmet0142 | *c*-type cytochrome, 8 hemes | Periplasmic | 0.14 | -0.71 ^**^ |
| Gmet0155 | *c*-type cytochrome, 1 heme | Periplasmic | -0.84 ^*^ | -1.86 ^***^ |
| Gmet0170 | *c*-type cytochrome, 1 heme | Periplasmic | 0.12 | -0.43 |
| Gmet0295 | *c*-type cytochrome nitrite reductase small subunit, 4 hemes | Cytoplasmic membrane | -1.13 ^*^ | -1.11 ^**^ |
| Gmet0328 | *c*-type cytochrome, 2 hemes | Periplasmic | -1.04 ^***^ | -2.08 ^***^ |
| Gmet0534 | *c*-type cytochrome, 5 hemes | Unknown | 1.46 ^**^ | 1.03 ^*^ |
| Gmet0557 | *c*-type cytochrome, 4 hemes | Extracellular | 0.24 | -1.20 ^***^ |
| Gmet0558 | *c*-type cytochrome, 27 hemes | Extracellular | 1.00 ^***^ | -0.37 |
| Gmet0571 | *c*-type cytochrome, 26 hemes | Extracellular/Periplasmic | 0.94 ^***^ | -0.81 ^**^ |
| Gmet0575 | *c*-type cytochrome, 18 hemes | Extracellular | 1.66 ^***^ | 0.30 |
| Gmet0576 | *c*-type cytochrome, 13 hemes | Extracellular | 0.81 ^**^ | 0.53 ^*^ |
| Gmet0580 | Lipoprotein *c*-type cytochrome, 14 hemes | Extracellular | 1.29 ^***^ | -0.44 |
| Gmet0581 | Lipoprotein *c*-type cytochrome, 27 hemes | Extracellular | 1.31 ^***^ | -0.49 ^*^ |
| Gmet0601 | *c*-type cytochrome, 8 hemes | Extracellular | -0.26 | 1.30 ^***^ |
| Gmet0679 | *c*-type cytochrome, 5 hemes | Unknown | -0.77 ^**^ | -0.61 ^*^ |
| Gmet0733 | *c*-type cytochrome, 1 heme | Outer membrane /Extracellular/Periplasmic | 1.46 ^***^ | -0.06 |
| Gmet0825 | *c*-type cytochrome, 12 hemes | Periplasmic | 0.37 | 0.32 |
| Gmet0827 | *c*-type cytochrome, 5 hemes | Extracellular | 0.50 | 1.12 ^*^ |
| Gmet0828 | *c*-type cytochrome, 6 hemes | Cytoplasmic membrane /Periplasmic | 0.39 | -0.43 |
| Gmet0909 | *c*-type cytochrome, 9 hemes | Cytoplasmic membrane | 1.01 | 1.79 ^**^ |
| Gmet0910 | Lipoprotein *c*-type cytochrome, 10 hemes | Extracellular | 0.41 | 0.42 |
| Gmet0912 | *c*-type cytochrome, 8 hemes | Periplasmic | 0.34 | -0.05 |
| Gmet0913 | *c*-type cytochrome, 9 hemes | Extracellular | 0.40 | -0.22 |
| Gmet0930 | *c*-type cytochrome, 6 hemes | Extracellular/Periplasmic | -1.31 ^***^ | -0.11 |
| Gmet1087 | *c*-type cytochrome, 1 heme | Extracellular/Periplasmic | 1.37 | 2.01 ^**^ |
| Gmet1088 | Lipoprotein *c*-type cytochrome, 1 heme | Periplasmic | 0.89 ^**^ | 1.32 ^***^ |
| Gmet1191 | *c*-type cytochrome, 1 heme | Cytoplasmic membrane | 1.38 ^*^ | 1.06 |
| Gmet1703 | *c*-type cytochrome, 7 hemes | Periplasmic | 2.67 ^***^ | 3.11 ^***^ |
| Gmet1744 | *c*-type cytochrome, 10 hemes | Periplasmic | 0.52 | 1.77 ^***^ |
| Gmet1809 | *c*-type cytochrome, 5 hemes | Cytoplasmic membrane | 0.91 | 0.03 |
| Gmet1814 | *c*-type cytochrome, 1 heme | Periplasmic | 0.83 | 3.67 ^***^ |
| Gmet1866 | *c*-type cytochrome, 4 hemes | Unknown | 1.49 ^**^ | 0.56 |
| Gmet1867 | *c*-type cytochrome, 7 hemes | Cytoplasmic membrane/ Extracellular | 0.81 ^*^ | 0.89 ^**^ |
| Gmet1868 | *c*-type cytochrome, 4 hemes | Extracellular | 0.55 | -0.05 |
| Gmet2048 | *c*-type cytochrome, 12 hemes | Periplasmic | 0.77 ^***^ | 1.06 ^***^ |
| Gmet2470 | *c*-type cytochrome, 27 hemes | Extracellular/Periplasmic | 1.09 ^**^ | 0.06 |
| Gmet2896 | *c*-type cytochrome, 4 hemes | Extracellular | 0.19 | -1.53 ^***^ |
| Gmet2899 | *c*-type cytochrome, 9 hemes | Periplasmic | 1.03 ^**^ | 0.26 |
| Gmet2930 | *c*-type cytochrome, 11 hemes | Periplasmic | 0.56 | 1.22 ^***^ |
| Gmet3088 | c-type cytochrome, 8 hemes | Cytoplasmic | 2.90 ^***^ | 1.31 ^*^ |
| Gmet3091 | c-type cytochrome, 2 hemes | Periplasmic | 0.30 | 0.01 |
| Gmet3518 | *c*-type cytochrome, 4 hemes | Cytoplasmic | -0.95 ^***^ | -2.27 ^***^ |
| Gmet1399 | Type IV major pilin subunit, PilA-N | Cytoplasmic membrane | 0.24 | 1.40 ^***^ |

Table S2 Transcriptional changes of GEMM-I riboswitch-controlled genes in Gme-L and Gme-H as compared to Gme-C. FC, fold change. *, 0.01 < *P* < 0.05; **, 0.001 < *P* <0.01; ***, *P* < 0.001.

| **Riboswitch** | **Selectivity on second messenger** | | **Locus ID** | **Log_2_FC**  **(Gme-L vs Gme-C)** | **Log_2_FC**  **(Gme-H vs Gme-C)** | **Annotation** |
| --- | --- | --- | --- | --- | --- | --- |
|  | Nelson et al., 2015 | Kellenberger et al., 2015 |  |  |  |  |
| Gm0131 | cGAMP | Undetermined | Gmet0131 | 0.84 | 0.06 | YXWGXW repeat-containing protein |
| Gm0232 | cGAMP | cGAMP | Gmet0232 | 0.41 | 0.07 | hypothetical protein |
| Gm0241 | cGAMP | cGAMP | Gmet0241 | -0.27 | 1.04 | DUF4382 domain-containing protein |
| Gm0292 | cGAMP | Undetermined | Gmet0292 | 0.21 | 0.44 | hypothetical protein |
| Gm0749 | c-di-GMP | cGAMP, c-di-GMP | Gmet0749 | 0.47 | 2.64 ^***^ | S8 family serine peptidase |
| Gm0751 | c-di-GMP | Undetermined | Gmet0751 | 0.93 ^***^ | -0.21 | S8 family serine peptidase |
| Gm0970 | cGAMP | cGAMP | Gmet0970 | -0.93 | 1.43 | helix-turn-helix transcriptional regulator |
|  |  |  | Gmet0971 | 0.79 ^**^ | 0.31 | type IV pilus assembly protein PilM |
|  |  |  | Gmet0972 | 0.75 ^*^ | 1.60 ^***^ | PilN domain-containing protein |
|  |  |  | Gmet0973 | -0.26 | -0.67 ^**^ | type IVa pilus biogenesis protein PilO |
|  |  |  | Gmet0974 | 0.18 | 0.72 | pilus assembly protein PilP |
|  |  |  | Gmet0975 | 0.23 | 0.76 ^***^ | type IV pilus secretin family protein PilQ |
|  |  |  | Gmet0976 | 0.10 | 0.17 | chorismate synthase |
|  |  |  | Gmet0977 | 0.37 | -0.42 | shikimate kinase |
|  |  |  | Gmet0978 | -0.04 | 0.46 | 3-dehydroquinate synthase |
| Gm1087 | cGAMP | cGAMP | Gmet1087 | 1.37 | 2.01 ^**^ | c-type cytochrome |
| Gm1191 | cGAMP | cGAMP | Gmet1191 | 1.38 ^*^ | 1.06 | c-type cytochrome |
| Gm1195 | cGAMP | cGAMP | Gmet1195 | 0.52 | 0.60 | S8 family peptidase |
| Gm1452 | c-di-GMP | Not tested | Gmet1452 | -1.12 | -1.76 | PEP-CTERM sorting domain-containing protein |
| Gm1703 | cGAMP | cGAMP | Gmet1703 | 2.67 ^***^ | 3.11 ^***^ | *c*-type cytochrome |
| Gm1763 | cGAMP | cGAMP | Gmet1763 | 0.93 ^**^ | 0.56 | hypothetical protein |
| Gm2037 | cGAMP | cGAMP | Gmet2037 | 0.43 | -0.36 | PEP-CTERM/exosortase system-associated acyltransferase |
| Gm2168 | cGAMP | Undetermined | Gmet2168 | 0.00 | 0.00 | VCBS repeat-containing protein |
| Gm3485 | cGAMP | cGAMP | Gmet3485 | 0.43 | 1.15 ^**^ | L, D-transpeptidase family protein |
|  |  |  | Gmet3486 | 0.39 | 1.82 ^***^ | Lpp/OprI family alanine-zipper lipoprotein |
|  |  |  | Gmet3487 | 0.20 | 1.66 ^*^ | L, D-transpeptidase family protein |

**Reference**

Nelson JW, Sudarsan N, Phillips GE, Stav S, Lünse CE, McCown PJ, Breaker RR. 2015. Control of bacterial exoelectrogenesis by c-AMP-GMP. Proc Natl Acad Sci U S A **112**:5389-5394.

Kellenberger CA, Wilson SC, Hickey SF, Gonzalez TL, Su Y, Hallberg ZF, Brewer TF, Iavarone AT, Carlson HK, Hsieh Y-F, Hammond MC. 2015. GEMM-I riboswitches from *Geobacter* sense the bacterial second messenger cyclic AMP-GMP. Proc Natl Acad Sci U S A **112:**5383-5388.

Table S3 Bacterial strains and plasmids used in this study.

| **Strain** | **Description** | **Source** |
| --- | --- | --- |
| *Geobacter metallireducens* | | |
| GS-15 | Wild type (WT) | ATCC^®^ 53774^TM^ |
| Gme-L | Wild type carrying pYhjH that decreases c-di-GMP level | This study |
| Gme-C | Wild type carrying the empty vector pYYDT | This study |
| Gme-H | Wild type carrying pYedQ that increases c-di-GMP level | This study |
| SL1 | Deletion of *Gmet0601* in strain GS-15 genome (contain Loxp site) | This study |
| SL2 | Deletion of *Gmet1703* in strain GS-15 genome (contain Loxp site) | This study |
| SL3 | Deletion of *Gmet1809* in strain GS-15 genome (contain Loxp site) | This study |
| SL4 | mutant Δ*Gmet0601* carrying complement pSL1 | This study |
| SL5 | mutant Δ*Gmet1703* carrying complement pSL2 | This study |
| SL6 | mutant Δ*Gmet1809* carrying complement pSL3 | This study |
| *Escherichia coli* | | |
| DH5α | Cloning host | TaKaRa Biomedical Technology (Beijing, China) |
| **Plasmid** | | |
| pYYDT | Km^R^; *oriV*(pBBR1), P*tac* | Lab stock |
| pYedQ | Overexpress *yedQ* gene | Lab stock |
| pYhjH | Overexpress *yhjH* gene | Lab stock |
| pUC19 | pUC19 carrying spectinomycin resistance cassette flanked by *loxP* sites | Lab stock |
| pCM158 | Cre recombinase expression vector; Km^R^ | Lab stock |
| pSL1 | The *Gmet0601* gene cloned in pYYDT | This study |
| pSL2 | The *Gmet1703* gene cloned in pYYDT | This study |
| pSL3 | The *Gmet1809* gene cloned in pYYDT | This study |

Table S4 Primers used for mutant construction and genotype validation.

| **Primer** | **Sequence (5' to 3')** | **Usage** |
| --- | --- | --- |
| YYDF | GCCTCAGGCATTTGAGAAGCACA | Validation of empty vector |
| YYD-R2 | TTGACGAGTTCTTCTGAGCG |  |
| 0601-upF | GAGCTCGGTACCCGGGGATCACCTACACCGTGACCCTGAC | Construction of Δ*Gmet0601* |
| 0601-upR | TTCCTTCCTCCTCGTTATTGTTCGTTTCTCCTTTCAGTTCCTG |  |
| 0601-dnF | TAGGTCCACCATGGCGGGGAGTCCTCTGGCCGGACAGTGA |  |
| 0601-dnR | CAGGTCGACTCTAGAGGATCAGGTTGGCGTAGAGACCGAAG |  |
| 0601-yz-F | TCTCCGTCACCAAGACCTAC | Validation of Δ*Gmet0601* |
| 0601-yz-R | TCAGCTCGCGGATAACCAG |  |
| 0601-hb-F | GGACTAGTCCAGTGAGACTAATCAGAGTCC | Coding sequence of Δ*Gmet0601* |
| 0601-hb-R | AACTGCAGAACCAATGCATTGGTCACTGTCCGGCCAGAGGAC |  |
| 1703-upF | GAGCTCGGTACCCGGGGATCACATCATCATCAGTGGAAGAAG | Construction of Δ*Gmet1703* |
| 1703-upR | TTCCTTCCTCCTCGTTATTGACTTCTACCTCCCTGATGG |  |
| 1703-dnF | TAGGTCCACCATGGCGGGGATAGTTCTAACTCGTGACGCAAG |  |
| 1703-dnR | CAGGTCGACTCTAGAGGATCAGGTGGGAAAAGGGTCATG |  |
| 1703-yz-F | ATGAGATGGTCCACAAGCAG | Validation of Δ*Gmet1703* |
| 1703-yz-R | AGATGCCATCGGACGCTTC |  |
| 1703-hb-F | GGACTAGTCCATATGGCAACCCGGCTGTT | Coding sequence of Δ*Gmet1703* |
| 1703-hb-R | AACTGCAGAACCAATGCATTGGCTTGCGTCACGAGTTAGAACTA |  |
| 1809-upF | GAGCTCGGTACCCGGGGATCCACAACGACGCGAGGAAATC | Construction of Δ*Gmet1809* |
| 1809-upR | TTCCTTCCTCCTCGTTATTGGCATCCACCTGAACGGGAG |  |
| 1809-dnF | TAGGTCCACCATGGCGGGGACGGAGCGCAAATGAAACGG |  |
| 1809-dnR | CAGGTCGACTCTAGAGGATCGCAGCAATCCGAAGCAAGTC |  |
| 1809-yz-F | accgagatttacaagacttggag | Validation of Δ*Gmet1809* |
| 1809-yz-R | cgtaatagggaagggtaggct |  |
| 1809-hb-F | GGACTAGTCCacgttccgccgtcaccgcaa | Coding sequence of Δ*Gmet1809* |
| 1809-hb-R | AACTGCAGAACCAATGCATTGGgttctccgtttcatttgcgctccg |  |

Table S5 The polypeptides used for antibody production.

| **Name** | **Molecular mass (kDa)** | **Polypeptides** |
| --- | --- | --- |
| PilA-N(Gme) | 7.8 | ESDLKNTKTNLESYYSEH |
| Gmet2896 | 25 | N-CHAVHGQTNTTYNYPKFLR |
| Gmet0913 | 54 | N-CLTSDEPGGYAHNRKYVKRL* |
| Gmet0825 | 34 | N-CTDCHGLPHSKQLHERFPK |
| DnaK | 68 | N-Cys-EKVVDADFEEVKDDKK* |

* Zhuo S, Jiang Y, Qi L, Hu Y, Jiang Z, Dong Y, Shi L. (2024) The robustness of porin-cytochrome gene clusters from *Geobacter metallireducens* in extracellular electron transfer. mBio, 15(9): e00580-00524.


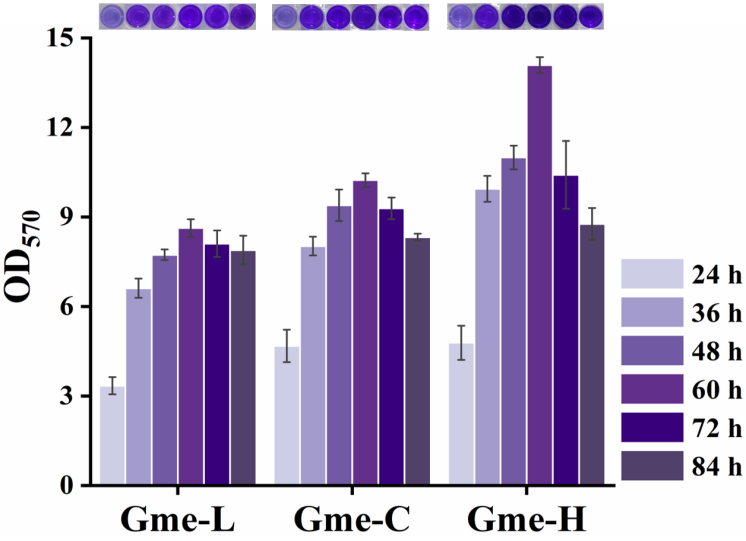


Figure S1 Crystal violet-stained biofilms grown in plate wells (upper panel) and the absorbance of biofilm extracts at 570 nm (lower panel). All results are reported as mean and standard deviation (n = 4).


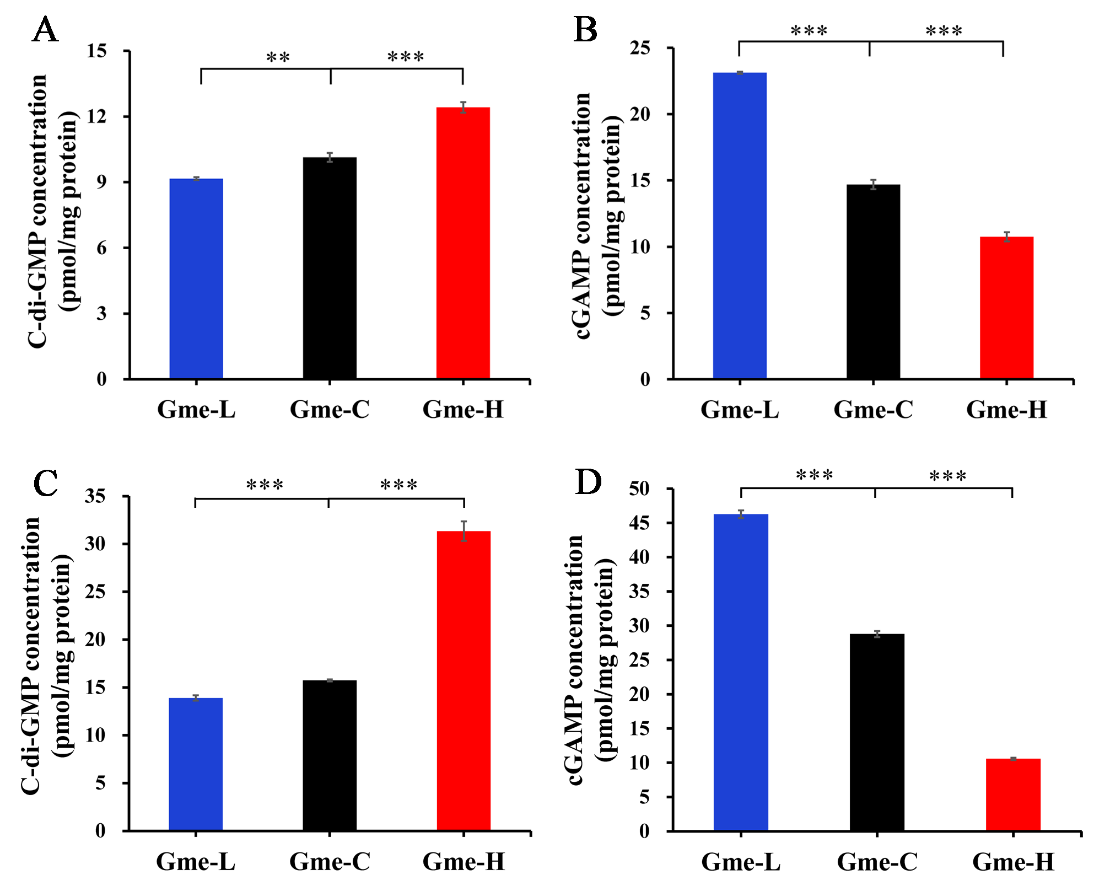


Figure S2 Intracellular levels of c-di-GMP (A, C) and cGAMP (B, D) in planktonic cells in anolyte (A, B) and biofilms grown on anode (C, D). All results are reported as mean and standard deviation (n = 3). **, 0.001 < *P* < 0.01, ***, *P* < 0.001.


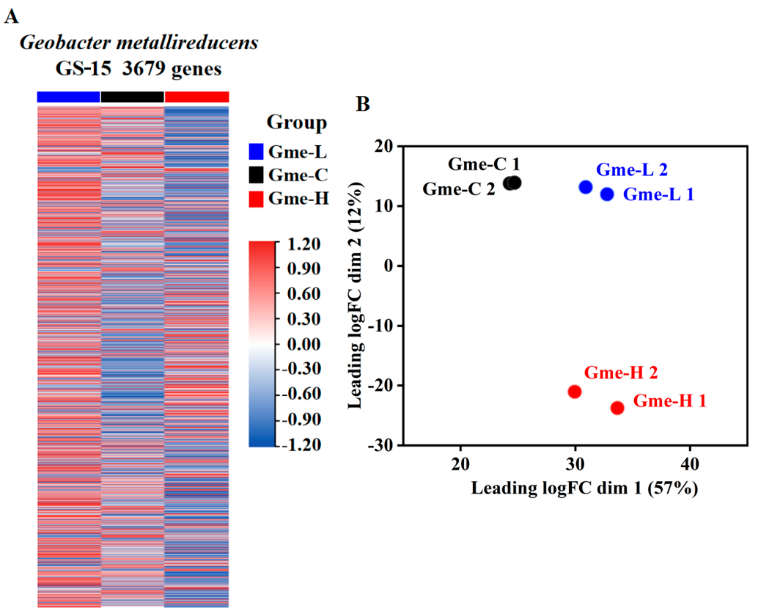


Figure S3 Transcriptome. (A) The transcriptome heatmaps of Gme-L, Gme-C and Gme-H biofilm cells. The biofilm cells on the anodes were collected when MFCs produced maximum current. (B) Multidimensional scaling plots of the gene expression of Gme-L, Gme-C and Gme-H biofilm cells.


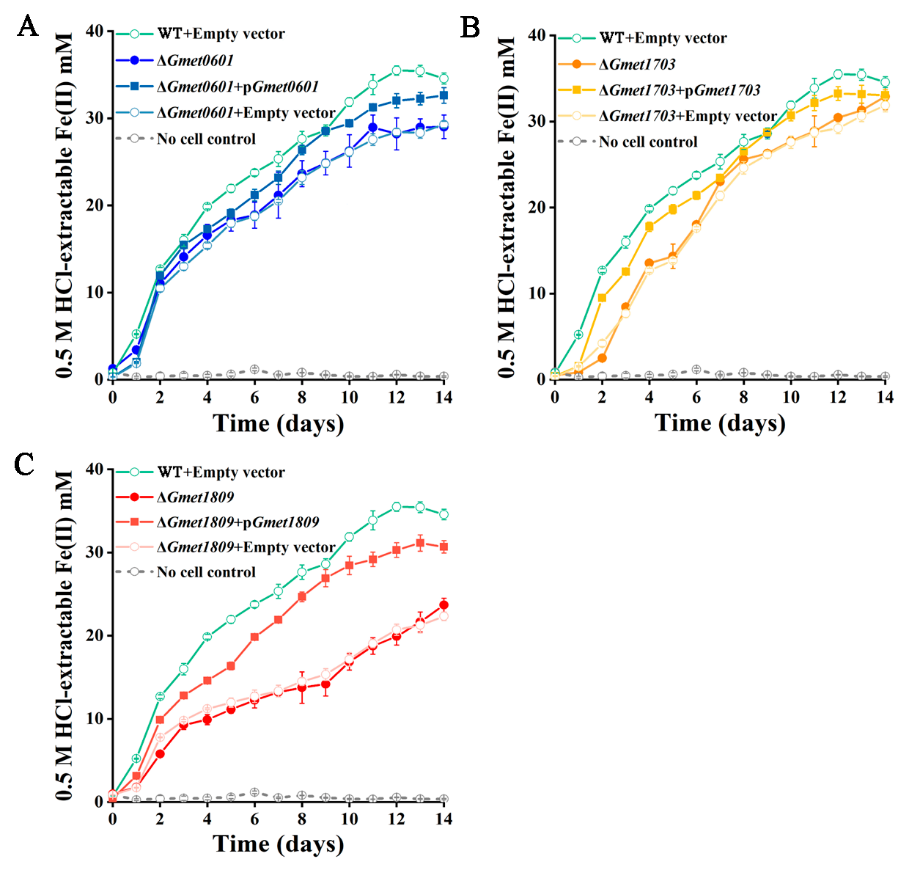


Figure S4 Ferrihydrite reduction by wild-type *G. metallireducens* GS-15 and mutant strains. Shown are the results within 14 days after reduction of ferrihydrite.


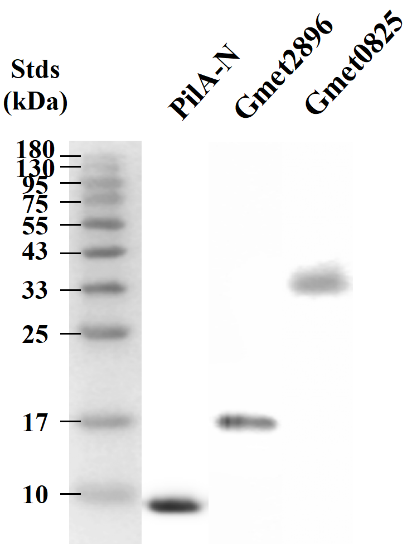


Figure S5 Immunoblot characterization of the produced antibodies specific for PilA-N, Gmet2896 and Gmet0825. Equal amounts of whole cell lysates were loaded to each lane of a 12% SDS-PAGE gel. The migration positions of standard proteins (Stds in kilodaltons or kDa) are shown at left.
